# Global externalities from avoided emissions in the Costa Rican cattle sector: opportunities for more efficient mitigation policies

**DOI:** 10.1007/s43621-021-00045-8

**Published:** 2021-08-11

**Authors:** Felipe Dall’Orsoletta, Andrei Domingues Cechin

**Affiliations:** 1 Environmental Economic Management, Center for Studies on Environmental and Agricultural Economics (CEEMA-UnB), University of Brasília, Brasília, DF 70910-900 Brazil; 2 Department of Economics, Center for Studies on Environmental and Agricultural Economics (CEEMA-UnB) and Center for Agroecological and Organic Production Studies (NEA-UnB), University of Brasília, DF 70910-900 Brasília, Brazil

## Abstract

The livestock sector has had an important contribution to global greenhouse gas (GHG) emissions. In Costa Rica, more than 20% of emissions come from beef and milk production. This paper performs a cost–benefit analysis of a climate policy in the Costa Rican cattle sector, and tries to innovate by including the positive global externality of emissions reduction into the analysis; to assess the extent to which it affects the attractivity of the referred policy. National sectorial policies for climate change mitigation generate global benefits, such as avoided GHG emissions into the atmosphere—a global public good. However, such global positive externalities, which represented 13% to 31% of the policy’s benefits in the widest scenario of our study, are usually not included in national climate planning, which may lead efficient policies to be dismissed. This paper shows that taking externalities into account makes sectorial climate mitigation policies more efficient, i.e., more appealing for investments. Benefit–cost ratios varied between 0.27 and 7.31 and break-even points average around the third and fourth years. Moreover, the results under different economic assumptions varied in terms of net benefits, but viability balance (viable vs. unviable scenarios) remained stable for different settings. The crucial question remains on how to best balance such global positive externalities to be advantageous to both funders and beneficiaries, enabling an efficient global climate mitigation strategy.

## Introduction

The main cause of climate threats perceived on our planet is the excessive generation of greenhouse gases (GHGs) into the atmosphere by anthropic activities [1]. Economic impacts coming from this causal relationship, when not fully internalized by GHG emitters, are considered an externality since they are not internalized into the cost functions of economic systems. Since the 1980s, societies have been looking for means to avoid these effects by driving economic systems to lower emission patterns without losing economic growth. Despite standing efforts, climate^1^ action worldwide is considered insufficient to avoid the most worrisome impacts [2].

Climate change (CC) presents some unique challenges that could be outlined under three overarching topics: impacts are felt in the world as a whole and under different intensities, some effects are nonlinear and involve a very long timeframe, and the reduction of GHG emissions is a global public good [3]. The last is because the atmosphere itself is classified as such (public good), i.e., its use has no rivalry and no exclusivity [4]. Therefore, any mitigation action can be considered as a generator of global environmental benefits [5] and, on purely economic grounds, it is in humanity's best interest [6].

Indeed, the livestock sector is likely to be one of the most complex and interlinked sectors for mitigation policies [7], and this may lead to situations where no country feels encouraged to carry CC mitigation policies, from the individual (national) economic rationality, since benefits will not be delimited to the funders of actions: they are shared globally [5, 8]. Put another way, national climate plans may not be (sufficiently) put into practice, as costs are often confined to a singular (or a group of) agent(s), while other countries would be receiving positive externalities from the interventions [1, 9, 10] This might be the essence of the problem, well characterized by the United Nations’ Secretary General in the context of the COVID-19 pandemic: “(…) Like the coronavirus, GHG respect no boundaries. Isolation is a trap and no country can succeed alone.^2^”

There is a growing body of literature on the contribution of the livestock sector to GHG emissions [11, 12]; all of which broadly conclude that livestock products are more GHG intensive than other food groups. Livestock production contributes to CC mostly through emissions of methane and nitrous oxide. The sector presents a substantial mitigation potential, even when considering the heterogeneity of its production systems [13].

Among the global continental regions, Latin America and the Caribbean are responsible for the highest share of cattle GHG emissions in the world [14], and in Costa Rica, beef and milk production generates over 20% of national emissions [15]. This paper performs a cost–benefit analysis (CBA) of a climate policy in the cattle sector of the country, with the inclusion of the global positive externality from emissions reduction. We assess the relevance of such benefits for the attractivity of this Costa Rican climate policy; and our hypothesis is that this economic valuation (of climate policies’ carbon-balances) can increase the societal profitability of such actions [5].

In Sect. 2, we briefly present the conceptual foundation and the issue of mitigation in the livestock sector, where our empirical assessment is justified. Section 3 outlines our empirical strategy, specifying the assumptions, scenarios and data collection. The results are presented in Sect. 4 in four different CBA rounds. Finally, we discuss the implications of the paper’s main findings in Sect. 5 before the conclusion.

## Literature review

### Social cost of carbon

The social cost of carbon (SCC) is a pricing tool commonly used in CC economics to estimate the economic benefits of GHG emissions reduction [4, 10, 16, 17]. It represents the present value of the external marginal damage of an additional unit of CO_2_ emitted, which includes impacts on inter alia, agricultural productivity and human health, infrastructure damages due to sea level rise and extreme weather events, and losses of biodiversity and ecosystem services [18]. Worth to say, as quite a complex task with substantial pieces of uncertainties and speculation; prudence is always required on its interpretations [17]. The range of SCCs’ estimates in the literature reflects the uncertainties of our understanding about CC and its future socioeconomic variables and the contrasting adopted ethical value judgments, particularly on how to aggregate values across time. The discount rate is probably the most important driver of this variability [19].

William Nordhaus, one of the pioneers of the field [20], perceives CC as a separated interactive module of the economic system [3, 16], where economic activities increase GHG concentration, which, in turn, causes damage to the economy. His main pursuit is to find the most efficient resource allocation strategy under such conditions, so mitigation policies that reduce GHGs at a per-unit cost lower than the SCC would pass a benefit–cost test. In fact, most current analyses use “integrated assessment models” (IAMs), following Nordhaus’ basic methodological approach. The purpose of these models is to determine the optimum reduction in GHG emissions—and the timing for that—in the face of CC damage. The reasoning is that climate mitigation ought to give a better rate of return than the market. Therefore, he uses market-observed discount rates within his studies. The most recent Nordhaus’ SCC estimation for such an optimum scenario [16] resulted in a value of USD 30 per tonne of CO_2_ (t/C).

On the other side of the spectrum is Nicholas Stern [17], who bases his parameters on two main premises. First, a prompt reaction would unleash a series of positive countereffects, enough to control the climate hazards and compensate eventual losses from such a rushed transition. Second, future generations deserve the same weight of importance as present ones. A high discount rate suggests that those alive today are worth more than future generations, entailing that societies should act like a single, self-regarding, optimizing, infinitely lived agent [21]. Hence, Stern suggests a discount rate much close to zero (it is not zero only due to the odds of a human life vanishment) [17]. Consequently, Stern’s model results in higher SCCs and drives more attention to the urgency of the climate crisis [22]. The economist estimated an ideal SCC at USD 85/tC for the business-as-usual (BAU) scenario. However, his analysis forecasts discounted losses from CC in a BAU scenario of 5% to 25% of world GDP while asserting that the discounted cost of stabilizing GHG at 550 parts per million of CO_2_e would cost approximately 1% of world GDP in 2050. Regarding these conclusions, Tol [23] notes that “Compared to the peer-reviewed literature, Stern’s estimate lies beyond the 95th percentile—that is, it is an outlier”.

Robert Pindyck, on the other hand, states that “IAMs cannot tell us anything about catastrophic outcomes, and thus cannot provide meaningful estimates of the SCC” [[24] (p. 14)]. He is critical about a supposedly excessive level of arbitrariness of climate models and their SCCs, stressing yet inadequate approaches to the likelihoods of extreme event occurrence. The economist stands for a more subtle and coherent gradation of variables regarding their causes and effects relations [25]. His input variables were built from a survey with hundreds of specialists regarding their opinion on the probabilities of alternative economic outcomes of CC and the SCC required to reduce emissions enough to avert such consequences. From such a venture, an SCC of USD 80/tC was defined as the best guess outcome [24].

### The livestock sector and the climate change

As climate debates gain intensity, more relevant become the agriculture and cattle sectors into the global arena [26, 27] due to their relevance within the global GHG matrix, their potential for CO_2_ mitigation and capture, and global food security issues, inter alia [28, 29].

There is much uncertainty regarding livestock’s contribution to global GHG emissions. Some try to estimate it as being between 10 and 12%, often highlighting the fact that the sector is the largest non-CO_2_ gas emitter [29].

Agricultural emissions are expected to experience major increases for the next decade, in contrast to other sectors that show bolder efforts to reduce their emissions [29]. Nevertheless, cattle activities can be allies of climate action, given their potential to mitigate emissions and capture CO_2_ from the atmosphere. The two main mitigation processes in the cattle sector are increases in productivity and operational improvements. The former could be summed up in genetic and nutritional advances; the latter is wider in scope and can involve different stages of the productive chain (pasture and manure management and land use, to name a few) [13, 30].

The sector’s potential for GHG mitigation and carbon capture comprises multiple assumptions, with different estimations and still many uncertainties [13]. Gerber et al. [13] estimated that livestock emissions could be 30% lower if all producers followed the practices of the 10% less intensive farms. Regarding carbon capture, the United Nations Food and Agriculture Organization (FAO) [31] stated that sound practices could capture some 600 million tonnes (t) of CO_2_eq per year, while Garnett et al. [30] estimated this amount as being between 295 and 800 million t, some 20–60% of the sectorial emissions.

However, choosing the best policy approach is not only about these hypothetical potentials but also about financial viability. Webb et al. [32] show that financial optimization (under a GHG reduction scenario) of the livestock chain in the United Kingdom would require a drop of over 50% of Britain’s beef production. However, the authors agree that such a reduction would not be reasonable for a single segment due to the likelihood of worse economic rebounds. Therefore, the crucial (and tough) challenge is to reach the required targets under an adequate balance between reduction and capture while maintaining the output capacity and the financial sustainability of the sector.

Henderson et al. [33] estimated the costs of reducing emissions from ruminant livestock using five different practices and showed that much of the total potential is not attainable in a cost-effective manner. Combined, the abatement options were estimated to save 11% of annual ruminant GHG emissions globally. Indeed, proposals to reduce GHG emissions that present a relatively high upfront cost may prevent farmers from adopting them without proper funding. Henderson et al. argue that stronger policy options, such as a carbon tax or emission quotas, would be required for costlier abatement options. The reason for this is that even if mitigation proposals are viable when positive global externalities of GHG emissions reduction are included in the financial assessment, costs are borne by farmers and national governments, while a considerable part of benefits is shared globally.

### The livestock sector in Costa Rica and its climate change mitigation policy

The cattle sector generates about 1.6 Gt of CO_2_eq each year in Latin America and the Caribbean, approximately 35% of global cattle emissions [34]. In this context, Costa Rica has been an active player in both environmental and climate engagement, with ambitious targets such as being the first carbon neutral country of the world in 2021 [35, 36]. Within the Paris Agreement, the country has committed to reducing 44% of its GHG emissions by 2050 (2012 as baseline).^3^

The cattle chain is responsible for over 20% of GHG emissions in Costa Rica, with herds occupying one-fifth of the national territory [37, 38]. The demand for these goods is expected to increase in the next decades, meaning that technical and operational improvements will be required both to meet these new needs and to respect the Paris Agreement pledges [35]. This is the purpose of programs such as the Nationally Appropriate Mitigation Actions (NAMA) conducted in the country.

The program is based on three main lines of action: reduction of methane emissions from enteric fermentation, reduction of methane and nitrous oxide emissions from manure management, and increases in the lands’ carbon capture potential. For these purposes, five mitigation measures were selected—rational grazing, use of hedges, better selection of pastures, and enhanced fertilization from manure management and reforestation—divided into four stages of implementation. Basically, the only difference from one phase to another was the number of farms involved, as initial phases were supposed to serve as a kind of laboratory for the program’s expansion (in the subsequent steps). Thereby, while the two initial periods were scheduled to last four years and aimed to reach less than 100 farms each one, phases 3 and 4 planned to reach 1,800 and 10,140 farms, in five and 10 years, respectively.

The project, that started in 2014, had a potential to mitigate approximately 4 million t of CO_2_eq. during its activities [37, 38], which could represent almost half of the efforts required by Costa Rica’s commitments under the Paris Agreement. Steps 1 and 2 required over USD 3 million of investments (comprising initial and operational costs) to be implemented and were supported by governmental and Agri-related institutions.

The third phase, however, would require investments up to USD 30 million—3.3 million of operational costs and 28,6 million of capital/technological investments—and the same actors did not show willingness or capacity to maintain their commitments to the disbursements, even in the face of potential financial gains coming from the interventions (all selected measures presented positive net present values (NPVs) and internal rates of return of over 10%). Despite good private perspectives, the continuity of the plan was under threat, and the leap from initial to last stages stood as a challenge for the planners at that time.

An assessment to gauge the financial viability of this subsequent stage suggested promising indicators for the program’s continuance: NPVs ranged from USD 425 to almost USD 15,000/farm, and internal rates varied between 22 and 492% [33] (for further details see Appendix 1). Such a prediction makes one question the reasons why investments have been considered so challenging, to the extent of possibly hindering the policy’s continuation. Elevated initial costs, lack of technical knowledge, and high fees and transaction costs are some of the features that could help to explain such controversy. With this in mind, the inclusion of positive global externalities (from emissions reduction) in the policy’s CBA may make the program more attractive for investments.

## Materials and methods

### Cost benefit analysis: public and social

CBAs can be useful to address issues of efficiency, with the caution of understanding it as part of a broader decision-making process [10]. They can summarize many of the expected impacts to a common (monetary) base, allowing a clear distinction between the aggregate pros and cons of a venture. This tool has been used in the climate context since the 1970s, and more recently, its usage is based on weighting the costs of action against costs of inaction from a social welfare perspective [20]. Future consumption is compared to present levels, usually represented as a portion of GDP, that would be under threat if CC damage is not avoided.

A CBA can have its NPV represented by Eq. 1, where ∑ is the sum of net benefits (*Bt*—*Ct*) for each year (*t*), brought to the present by the discount rate (*r*). Such an indicator represents the present value of all benefits minus the present value of all costs (Eq. 2). From this, a second indicator can be derived, dividing the net present benefits, the present value (PV(*B*)), for the costs (PV(*C*)), to find the *B*/*C* ratio (Eq. 3), where a value higher than one suggests a viable scenario, and the higher it is, the more efficient the project is supposed to be.

1 $${\text{NPV }} = \, \sum_{{{\text{t}} = 0}}^{{\text{T}}} \left( {B_{{\text{t}}} - C_{t} } \right) \, / \, \left( {{1} + r} \right)^{t}$$

2 $${\text{NPV }} = {\text{ PV}}\left( B \right) \, {-}{\text{ PV}}\left( C \right)$$

3 $$B/C = {\text{ PV}}\left( B \right) \, /{\text{ PV}}\left( C \right)$$

According to Pigou [39], there are private costs and benefits, and external costs and benefits. Social costs and benefits are the sum of private and external costs and benefits. Therefore, divergences between private and social costs and benefits can be identified as externalities. When a cattle farm renders a benefit of a service to other agents without appropriating to itself all the benefits of his service—for example, in the form of reduced future environmental costs—it is an external benefit. That is, when social benefits exceed private benefits, it is a positive externality.

This CBA decomposes costs into public and private, while benefits are decomposed into private and external. Public costs are disbursements borne by the public budget, exclusively regarding initial investments, while private costs include both the farmers’ operational costs and their expenses with initial investments. To make it a more realistic scenario, we emulate a financial market situation under analogous contexts. Money-lending companies often provide between 70 and 80% of the total amounts claimed by climate-related projects [40], so we chose the mean value (75%) as being the amount entailed to public funding—the rest is assumed to be left for farmers (or any other nongovernmental actor) to pay, leaning on the positive private perspectives. Besides the private operational gains of farmers, private benefits, there are positive global externalities resulting from expected avoided emissions,^4^ external benefits [42–44].

CBAs are named public and social (Table 1). Public CBAs regard exclusively nonprivate costs and benefits, that is, public costs with initial investments and the global positive externality of avoided emissions (external benefits). On the other hand, social CBAs aim to comprise all the forecasted costs and benefits coming from the program, i.e., private costs (investment and operational ones) and opportunity costs are aggregated to the public cost; whereas the private operational gains are also added to the external benefits (global positive externality of avoided emissions). The reason for decomposing the CBAs into a partial analysis, which considers only nonprivate costs and benefits, and a total analyses, which considers the totality of costs and benefits (private, public and external), is twofold: (i) to assess the economic viability of a “pure” environmental policy, with no consideration for private costs and benefits, and (ii) to make explicit the difference in viability when comparing the “pure” environmental policy with a sectorial policy, with private costs and gains, besides important climate implications.

**Table 1:** Cost and benefits considered for each CBA

CBA	Costs		Benefits	
	Initial investments	Operational costs	Avoided emissions	Operational gains
Public	Public (75%)	–	External	–
Social	Public (75%) + private (25%)	Private	External	Private

**Table 2:** The three sets of economic assumptions for the CBAs

Paper	Discount rate	The social cost of carbon
Nordhaus (2018) [16]	4,25%	30
Stern (2006) [17]	0,1%	85
Pindyck (2019) [44]	3%	80

SCC in US Dollars as in the original publications. Pindyck and Stern’s SCC are the best estimates.

Aiming to enhance the robustness of the analysis, we used three different sets of economic assumptions based on three published papers from the SCC literature: Nordhaus (2018), Pindyck (2019) and Stern (2006) [16, 17, 45] (Table 2).

### Data collection

Data about the NAMA program and its economic predictions were extracted from an internal NAMA Program Database.^5^ Only the third phase is considered in our analysis. It is assumed the full implementation of measures under the most optimistic scenario, which forecasts the avoidance of 3.437.201 tons of CO_2_eq emissions.

Operational costs, capital investments and economic benefits of avoided emissions were initially calculated for each farm, on average, and then aggregated for the total number of farms and for both time periods (5 and 15 years). CBAs were settled exclusively within the NAMA’s third phase features (Table 3), regardless of the implementation of the fourth phase.

**Table 3:** Basic inputs of CBAs

Variable	Specification	Input
Time period	–	5 and 15 years
Total no. of farms	–	1,800
*Costs*		
Total operational costs	–	$ 3,250,025a
Initial investments per farm (USD)	Meat	$ 16,629
	Milk	$ 4,822
	Mixed	$ 22,078
Initial investments per CBA	Public	$ 21,426,340
	Social	$ 31,818,478
*Benefits*		
Avoided emissions per farm (t/year)	Meat	68
	Milk	2.1
	Mixed	58.2
Avoided emissions benefits (per SCC)	Nordhaus	$ 5,808,254
	Stern	$ 16,456,719
	Pindyck	$ 15,488,677
Total operational gains	-	$ 37,942,679

Monetary values in USD. Based on Dall’Orsoletta [﻿^46^]

### Opportunity costs

The opportunity cost (OC) of a project regards the positive impacts that could have otherwise resulted from alternative scenarios, i.e., benefits that are being overlooked when choosing that project. In climate affairs, a common practice is to consider the nonpolicy scenario, where the OC is what societies should give up executing the plans.

Applying this logic to a specific sectorial climate policy (as the NAMA) is not that simple [47]. In the present case, the non-policy situation would probably mean the *status quo* for farmers; they should maintain their activities at the same (previous) productive level, and no tangible economic or environmental losses would be felt. From the social side, losses would probably attend to the deterioration of air quality and elevation of CC threats; however, given the atmosphere’s global feature, local-specific consequences would be marginal. Nonetheless, the public budget would present a substantial OC, as a society could choose any other destiny for the USD 30 million. Public resources could be invested in the renewable energy sector or in payment for ecosystem services, making sense for a wide climatic approach [48] but means nothing to the addressed livestock emissions situation.

In this paper, the initial CBA scenarios ignored the OC. However, considering such features for climate policies is of utmost importance [9], either for accountability purposes or for attending to counterarguments [47]. We chose two of the most cost-effective mitigation measures (not included in NAMA) for the large ruminant sector as our OC parameters to acknowledge the counterarguments. Alternatives were increased concentrate feedings in the animals’ food and biogas generation plants from methane emissions; feed additives also showed a good capacity for mitigation but were discarded due to prohibitive initial costs [38]. The two selected technologies were harmonized through a linear function to fit into monetary and physical NAMA features, allowing their equanimous aggregation into the analyses (details on Appendix 2). OCs were included only in Social CBAs, as their indicators presented no specification regarding private and public dimensions (for a detailed table with calculations for one scenario, see Appendix 3).

### Break-even years

The break-even period of an investment refers to the point (in time) where the present value of benefits become equal to the present value of costs. It is a useful indication of the viability of the implementation of mitigation measures, as lower break-even years indicate a more attractive scenario for investments. Moreover, it is also a signal of robustness of investments in relation to the chances of decrease in the policy’s adoption rate [5] or hypothetical desertions.

Brown et al. [6] ascertain that naturally endowed countries are likely to benefit earlier from climate change mitigation policies, depending on the national climate and economic backgrounds. To verify this and as a further evaluation metric for our analyses, in the last round we run a break-even analysis for each of the proposed CBAs.

Table 4 shows the whole set of scenarios (with and without OC) for the four different rounds.

**Table 4:** Settings of the 18 CBAs under the four rounds of scenarios, with CBA type, costs, benefits and economic assumptions

Round	Scenario	Type	Costs	Benefits	Economic assumptions	Discount Rate	Social Cost of Carbon (USD)
1—CBAs 5 years	PN 5	Public	75%	AE	Nordhaus	4.25%	30
	PS 5	Public	75%	AE	Stern	0.1%	85
	PP 5	Public	75%	AE	Pindyck	3%	80
	SN 5	Social	100%	AE + PB	Nordhaus	4.25%	30
	SS 5	Social	100%	AE + PB	Stern	0.1%	85
	SP 5	Social	100%	AE + PB	Pindyck	3%	80
2—CBAs 15 years	PN 15	Public	75%	AE	Nordhaus	4.25%	30
	PS 15	Public	75%	AE	Stern	0.1%	85
	PP 15	Public	75%	AE	Pindyck	3%	80
	SN 15	Social	100%	AE + PB	Nordhaus	4.25%	30
	SS 15	Social	100%	AE + PB	Stern	0.1%	85
	SP 15	Social	100%	AE + PB	Pindyck	3%	80
3—CBAs with OC	SN 5C	Social	100%	AE + PB	Nordhaus	4.25%	30
	SS 5C	Social	100%	AE + PB	Stern	0.1%	85
	SP 5C	Social	100%	AE + PB	Pindyck	3%	80
	SN 15C	Social	100%	AE + PB	Nordhaus	4.25%	30
	SS 15C	Social	100%	AE + PB	Stern	0.1%	85
	SP 15C	Social	100%	AE + PB	Pindyck	3%	80
4—Break-even years (for all scenarios)							

The first letter of the scenarios, P or S, represent Public and Social assumptions. In the second letter, N, S or P refer to Nordhaus, Stern and Pindyck. The number 5 or 15 indicates the timeframe in years, and letter C denotes cases where opportunity costs are included. As per the benefits, AE means avoided emissions and, PB, private benefits

## Results

### Round 1—Public and Social CBAs, 5 years

The first round showed equally divided results (Table 5). All the Public CBAs produced benefit-cost (B/C) ratios lower than one (ad hoc a negative scenario), while social B/C ratios were positive.

**Table 5:** Cost/benefit and the net present value of the first round of analyses: CBAs 5 years

Scenario	Benefit/ cost	Net present value (USD millions)
PN 5	0.27	− 13.9
PS 5	0.77	− 5.0
PP 5	0.72	− 5.5
SN 5	1.36	10.2
SS 5	1.71	22.5
SP 5	1.67	19.5

The highest value was found in SS 5 (1,71), and the lowest was in PN 5 (0,27). Net present values (NPVs) ranged from negative USD 14 million to positive USD 22 million. Adding private benefits to positive global externalities led to more attractive scenarios. All B/C relations more than doubled under such a social perspective, with major differences between Public and Social analyses with Nordhaus’ SCC and discount values.

**Fig. 1:**
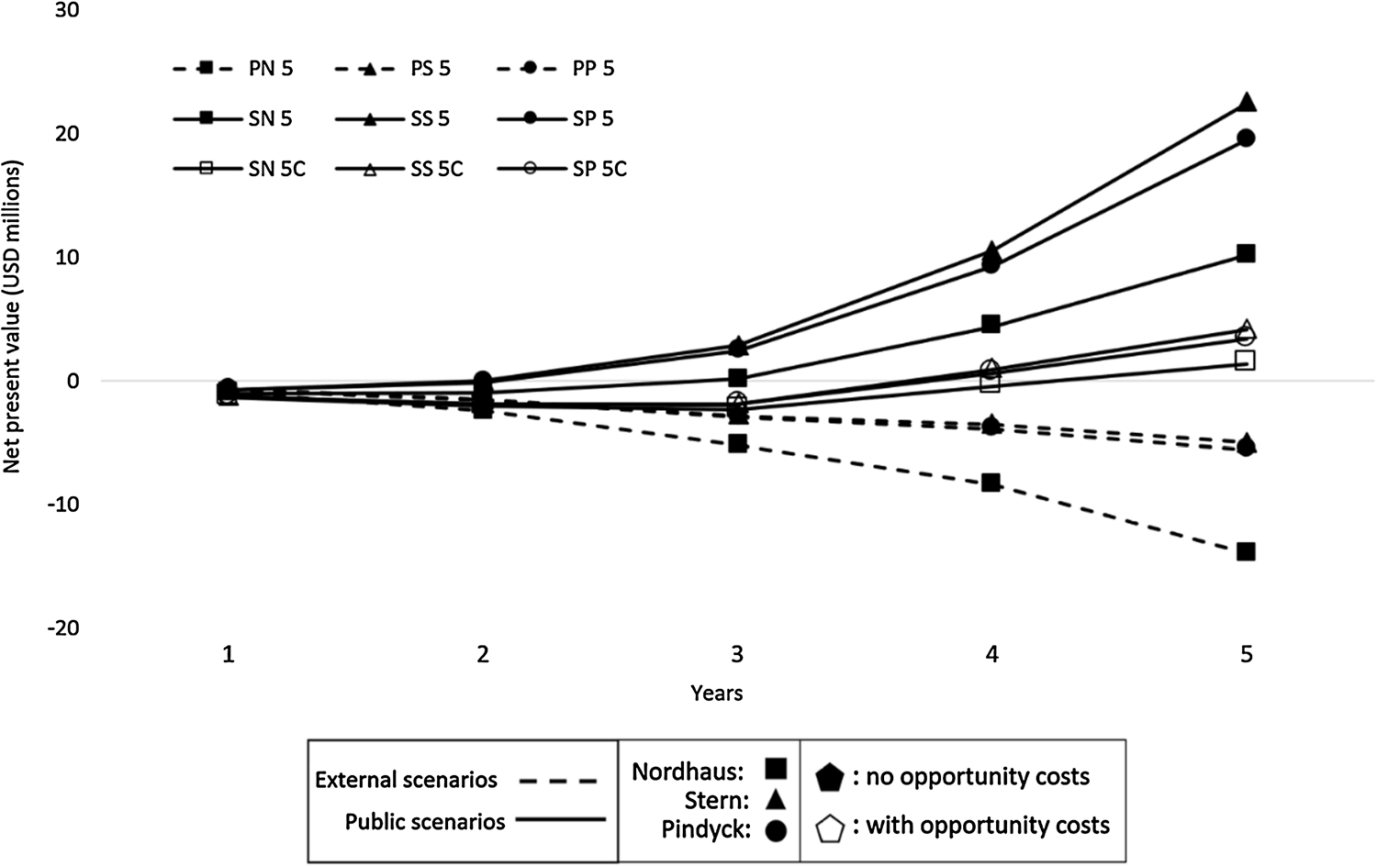
Accumulated net benefits curve of CBAs 5 years. Linear graph with 9 lines representing the Net Present Value of the nine 5-year scenarios, with the Net Present Value in the axis Y, and years (1 to 5) in axis X. The six Social Scenarios end up with positive Net Present Values, while the three Public Scenarios result in negative net values at the end of the fifth year

The curve of accumulated net benefits (ANB) (Fig. 1) shows that the highest volumes of benefits are present in the social analyses. Moreover, trends indicate that gaps will continue to increase, i.e., negative scenarios would tend to worsen as time passes, as indicated by the second round of the 15-year CBA.

### Round 2—Public and Social CBAs, 15 years

Outcomes of the scenarios with a wider time frame show different patterns (Table 6).

**Table 6:** Cost/benefit and the net present value of the second round of analyses: CBAs 15 years

Scenario	Benefit/ cost	Net present value (USD millions)
PN 15	1.27	5.1
PS 15	4.45	73.7
PP 15	3.6	51.1
SN 15	5	141.3
SS 15	7.31	264.8
SP 15	6.43	201.4

All B/C ratios were above one, with SS 15 and PN 15 again generating extreme results of 7,31 and 1,27, respectively. Concomitantly, NPV ranged between USD 5 and 265 million.

**Fig. 2:**
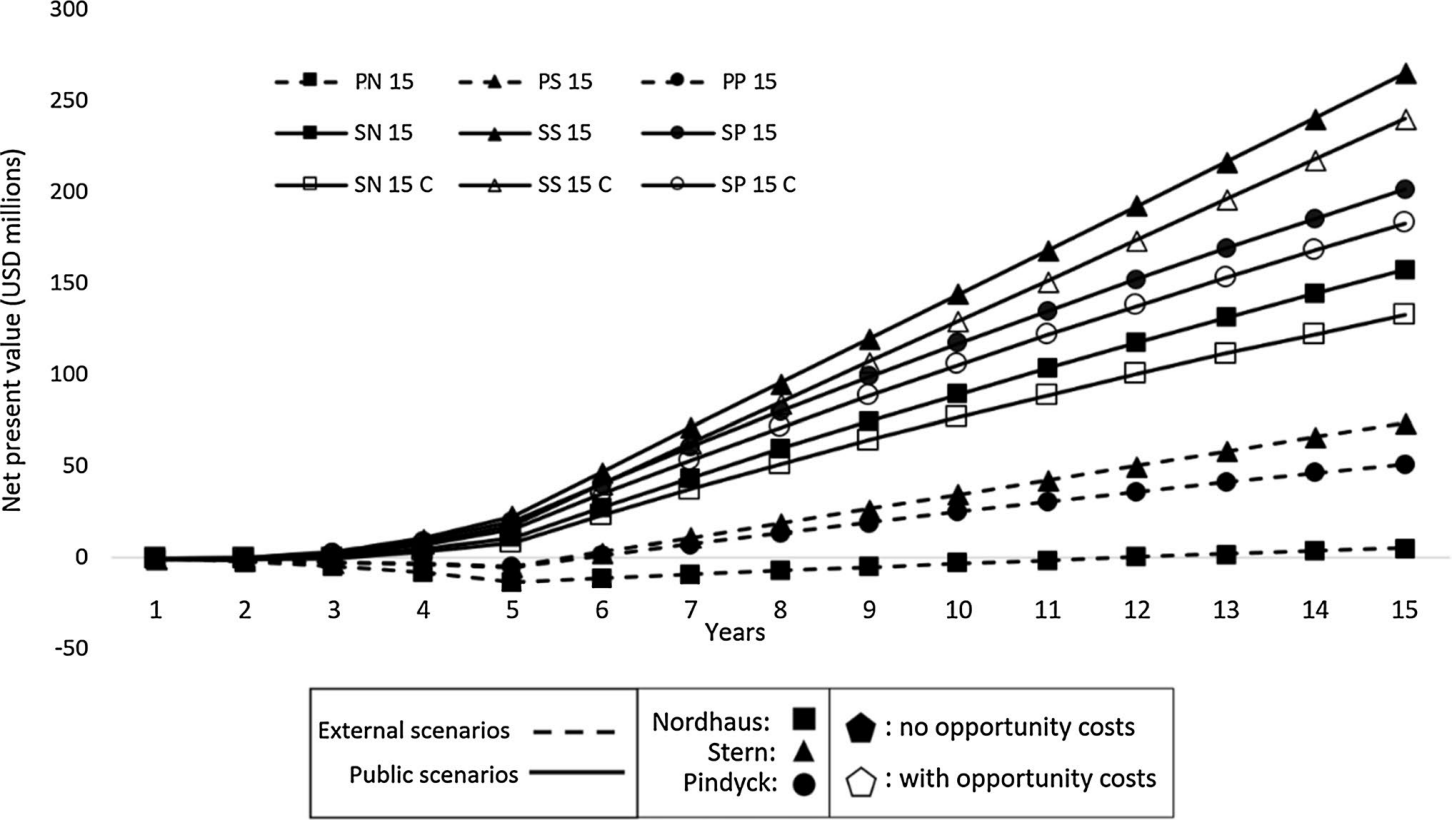
Accumulated net benefits curve of CBAs 15 years. Linear graph with 9 lines representing the Net Present Value of the nine 15-year scenarios, with the Net Present Value in the axis Y, and years (1 to 5) in axis X. All scenario result in positive Net Present Values at the end of the period, with the 6 Social scenarios consistently showing higher values

The 15-year ANB curve showed that the previous assumption of viability worsening after the fifth period was wrong. Instead, all scenarios ended up positive, with the latest payback occurring in the 12th year (Fig. 2). These results suggest that benefits will continue to increase in the future.

### Round 3—Social CBAs with opportunity costs, 5 and 15 years

The inclusion of the OC did not change the signal for any CBA (Table 7), thus, scenarios continued to be appealing for investments. Higher variations (regarding scenarios with and without OC) were noticed in the 15-year scenarios. The 15-year ANB curves (Fig. 2) show that attractiveness steadily increases over time despite the inclusion of OC. The three scenarios would reach their paybacks before the fifth year.

**Table 7:** Cost/benefit and the net present value of the third round of analyses: CBAs 5 and 15 years, with opportunity costs

Scenario	Benefit/ cost	Net present value (USD millions)
SN 5C	1.04	1.4
SS 5C	1.08	4.2
SP 5C	1.08	3.4
SN 15C	4	132.5
SS 15C	4.61	240.3
SP 15C	4.28	182.8

The highest B/C ratio (4.61) was that of scenario SS 15C, while scenario SN 5C presented the lowest (1.04). NPVs ranged between USD 1.4 and USD 4 million for the 5-year scenarios and between USD 132 and USD 240 million in the wider periods.

### Round 4—Break-even years

**Fig. 3:**
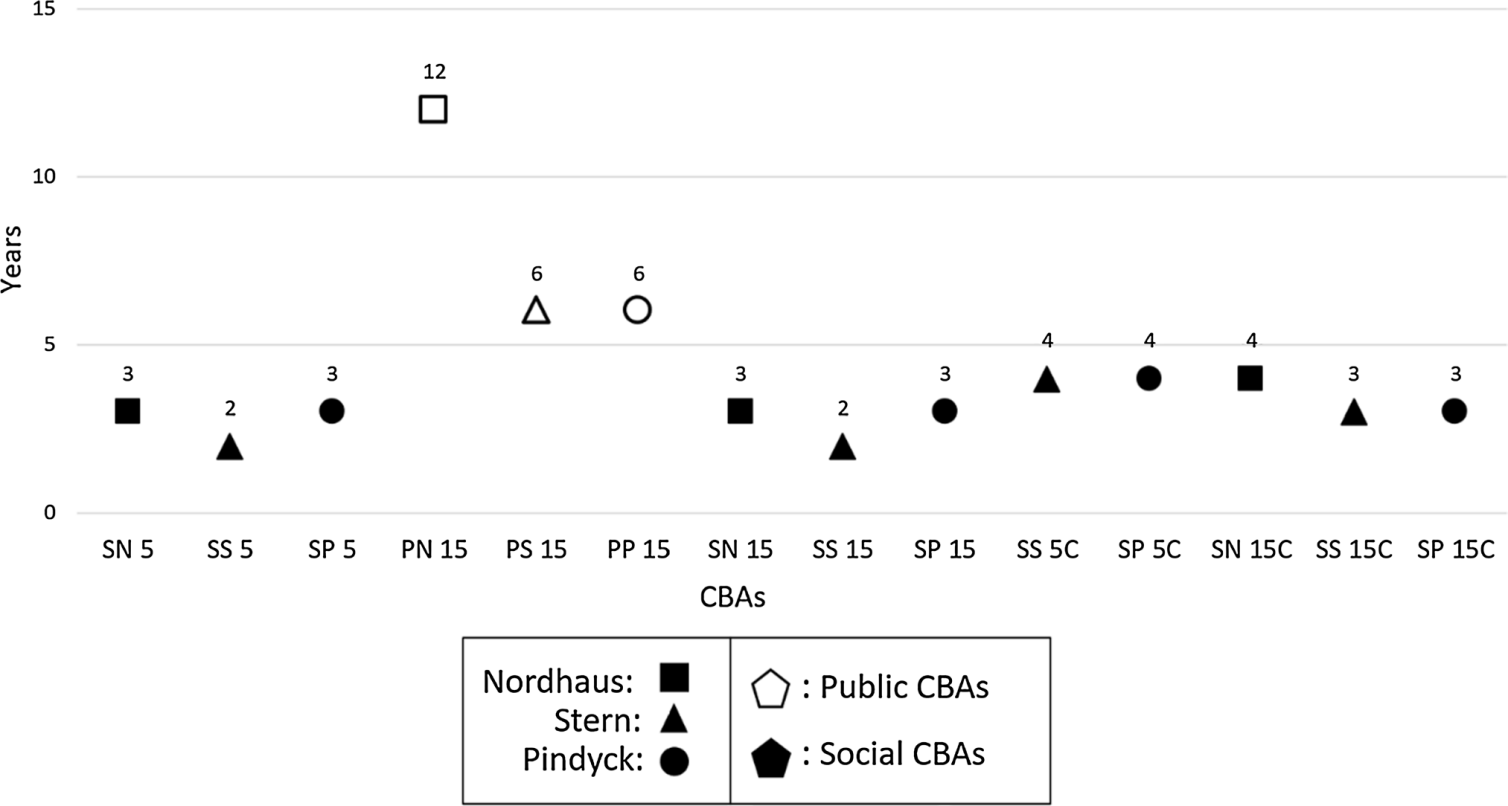
Break-even years of the CBAs. Dot plot graph representing the break-even years in which the investments would be neutralized, for each CBA. Axis X account for the years, running from 0 to 15, and axis Y shows the scenarios. All the Social CBAs record their pay off before the fifth year, whereas the resting three Public scenarios would reach their break-even point in years 6 and 12

Break-even years indicate how attractive is a viable investment plan, where lower break-even years indicate more attractive scenarios for implementation. In this regard, the NAMA program has achieved good indicators, as 11 out of the 18 scenarios registered a break-even point of four or less years (Fig. 3). All such lower break-even periods exactly refer to the Social scenarios and would fit into time-lapse of the NAMA’s third phase. From the other three CBAs where a break-even was possible, two would have their balance achieved on the sixth year, while the remaining one would require 12 years to achieve its monetary balance. Other four scenarios—all 5-years situations, one Social, three Public—would not achieve their profitability threshold within their time-lapses.

To assess break-even periods of Social against Public costs and benefits, we may look to the 15-year scenarios with no OC (it is the only situation where a same amount of Public and Social break-even points was generated). From such a perspective, the break-even means recorded 2.6 years for Social scenarios against 8 years for the Public ones. On average, the financial equilibrium point of all scenarios is somewhere between the third and fourth years (overall mode was 3).

### Compilation of results

From the 18 scenarios presented in rounds one, two, and three, 15 resulted in viable indicators (Table 8). Only the three 5-year scenarios of Public CBAs resulted in B/C relations below one. Values ranged between 7.31 and 0.27, with the best perspectives systematically being found in Social analyses under 15-year horizons. Amid the three economic references, Stern outlined better perspectives, while Nordhaus recorded the worst perspectives. Avoided emissions represented 13% to 31% of the total expected benefits in Social analyses, and 61% of the CBAs have their break-even point before the fifth year. Finally, the aggregation of OC lowered B/C ratios but not to the extent of reversing viability.

**Table 8:** Compilation of the 18 CBAs, in descending order according to the benefit cost ratio

Scenario	Benefit/cost	Net present value (USD millions)	Type	Time horizon (years)	Break-even years	Economic assumptions
SS 15	7.31	264.8	Social	15	2	Stern
SS 15C	4.61	240.3	Social	15	3	Stern
SP 15	6.43	201.4	Social	15	3	Pindyck
SP 15C	4.28	182.8	Social	15	3	Pindyck
SN 15	5	141.3	Social	15	3	Nordhaus
SN 15C	4	132.5	Social	15	4	Nordhaus
PS 15	4.45	73.7	Public	15	6	Stern
PP 15	3.6	51.1	Public	15	6	Pindyck
SS 5	1.71	22.5	Social	5	2	Stern
SP 5	1.67	19.5	Social	5	3	Pindyck
SN 5	1.36	10.2	Social	5	3	Nordhaus
PN 15	1.27	5.1	Public	15	12	Nordhaus
SS 5C	1.08	4.2	Social	5	4	Stern
SP 5C	1.08	3.4	Social	5	4	Pindyck
SN 5C	1.04	1.4	Social	5	–	Nordhaus
PS 5	0.77	− 5.0	Public	5	–	Stern
PP 5	0.72	− 5.5	Public	5	–	Pindyck
PN 5	0.27	− 13.9	Public	5	–	Nordhaus

## Discussion

The NAMA Program presents promising scenarios for its implementation, and the consideration of external costs and benefits only increased its viability. Its successful execution would represent 44% of the aimed GHG emissions reduction by the Costa Rican National Determined Contribution. Filho et al. [50] obtained values between 13 and 25% analysing different grazing intensities of steers in the soybean-beef cattle integrated system in southern Brazil. As a whole, NPVs stayed between negative USD 13.9 and positive USD 264.8 million. Per farm, values stayed between negative USD 7,722 and positive USD 147,127. Notwithstanding, the transition of livestock systems towards mitigation pathways are one of the most complex adjustments of the global food system [7], and therefore, these findings have to be seen in the light of some limitations.

The first is that costs and benefits do not necessarily pertain to a same agent. Therefore, even positive results (from a global perspective) could mean negative scenarios from an individual perspective (funders). A second limitation regards data inputs. This paper remained faithful to the NAMA Program database, despite the availability of more recent data, aiming to ensure a higher level of homogeneity and coherence of analysis. In the same sense, monetary values were calculated in United States dollars and always based on the original sources, with no deflation of values. Finally, alternative measures (used as OC) were taken from an Indian agricultural case,^6^ which possibly holds considerable differences when compared to the Costa Rican reality.

Considering only the 15-year scenarios (for a more analogous comparison with the NAMA Program framework), we find NPVs ranging from USD 2,843 to 147,127 per farm, against a range of USD 425 to 14,714, which resulted from the previous private financial assessment [46]. As different indicators and scenarios result in different outputs, we also compared the average NPVs per farm for both studies and found that our average value was over 20 times higher—USD 80,800 against 3,500 per farm NAMA Program database. Despite methodological unevenness, such a disparity suggests that positive global externalities represent a relevant share of the benefits of sectoral climate policies, in line with Branca et al. [5] findings.

B/C ratios varied between 0.27 and 7.31, and in the Social scenarios, positive global externalities represented 13% to 31% of the policy’s economic benefits. In a similar line, Branca et al. [5] concluded that the economic evaluation of carbon-balances in climate-smart agriculture investment plans could increase NPVs as of 1% to 7% under a trade-oriented scenario, and 95% to 700% in a more sustainable assumption, while Quinet and Brunel [8] showed that the potential for GHG emissions reduction could represent up to eight times the required investments of a project, reinforcing the notion that there is no rationality in ignoring such environmental features. By running a CBA for the European Union climate plan and considering the avoided emissions in the European territory as the only benefit, Tol [10] found B/C rates between 0.03 and 0.88. Would these rates be greater than one if other private and public benefits were considered? Bollen et al. [20] found, for example, that local environmental policies could unleash twice as many global climate benefits as local gains.

Quoting Tol [[10] (p.2)], “CBAs should always be interpreted with caution, because (…) they are never complete and rarely do justice to the complexity of situations”, whereas economic insights upon future costs and benefits of climate policies in the agricultural sector is a game-changing for the selection of the most economically efficient options [51]. In this context, while important to inform decisions on climate mitigation policies, efficiency cannot be its only criterion. Relying exclusively on such criteria may skew decisions towards insufficient mitigation efforts at present, especially due to discounting of future benefits of present mitigation efforts.

This important critical outlook on CBA, particularly on discounting future environmental benefits and costs, reveals some substantial implications of our findings. On the one hand, the outcomes in the many scenarios differ according to the set of economic assumptions and timeframe. On the other hand, a “viability balance” (number of viable and unviable scenarios) points out the same pattern—5 viable and 1 unviable—for each of the three main sets of assumptions (Nordhaus, Stern and Pindyck’s inputs). The only unviable scenarios were those considering exclusively external environmental benefits and under a short timeframe (5 years) The inefficiency of the three negative scenarios may be explained by the existence of significant initial costs, which, in the short term, are superior to the (discounted flow of) environmental benefits.

While the physical impacts of CC will be felt over a long-term horizon, with massive costs and possible civilizational impacts on future generations, the time horizon in which financial, economic and political players plan and act is much shorter. Therefore, the lifetime of climate policies and their effects must always be considered for the selection of the most appropriated method for a climate-economic appraisal [51]; and coherence between projects’ time frames and the nature of policies is necessary to overcome this “tragedy of the horizon” [52].

Mitigation policies such as the NAMA, which presented a relatively high upfront cost, might have prevented farmers and government from carrying on with their disbursement commitments—which is particularly relevant for developing nations that are often struggling to keep up with their public budgets and is also important to endorse this so-called global new economy (that incorporates social and environmental aspects on the same rate as the economic ones). Today, all-but 100% of ESG investments in the world come, take place or are managed in the northern hemisphere [53].

When including the global positive externalities of the policy’s avoided emissions, its economic assessment showed that the NAMA is viable in almost every scenario, despite the differences in economic assumptions. Break-even analyses walk in this same line, as most scenarios would demand relatively short intervals for their investments’ neutralization. Brown et al. [6] estimated the break-even point for the optimal implementation of global climate change mitigation policy and found that the offsetting of investments would be achieved after 60 years of implementation. Therefore, it can be inferred that, depending on this parameter, NAMA stakeholders would have a strong sign to invest in the program, and the odds of farmers abandoning the scheme would be lower—particularly if the (ad hoc) social approach is taken into account.

Thus, our results also suggest that the viability or unviability of policies may not be a matter of just raising or decreasing SCCs and social discount rates, but of presenting consistent and legitimate plans, particularly regarding the sharing of the mitigation costs burden. This goes in hand with Branca et al. [5], who consider that the economic evaluation of carbon-balance can justify the public financing of climate policies, while additionally helping to overcome other barriers such as the farmers’ resistance; and with Henderson et al. [33], for whom viable mitigation proposals in the cattle sector, with higher costs, may require economic incentive schemes—such as carbon tax or emission quotas—as a necessary condition for implementation.

It is also likely that private gains would be generated throughout the whole sector’s supply chain. The estimated indirect (intra- and interregional sectorial readjustments) mitigation benefits of a steady transition, from extensive to more productive systems, in the global cattle sector are twofold the direct benefits (best practices per se) [7]. From such additional externalities, further cascading effects may emerge, and thus green investments and the new economy tend to become more solid. Perhaps, a first step is international monetary and financial institutions start considering long-term climate stability as a global public good, so national economies could take these externalities into account when analysing the prospect of policies and asking for funding [52].

In this sense, although international and national institutions are far from achieving the structural change required for the aforementioned stability, some compensation schemes that acknowledge positive global externalities of avoided emissions are emerging. The Warsaw International Mechanism for Loss and Damage associated with Climate Change Impacts, for example, aims to balance climate-driven losses of developing countries with compensation from major emitters. In the same vein, the Carbon Offsetting and Reduction Scheme for International Aviation (CORSIA) may be the only emissions scheme with a common cap for the world’s carbon emissions. Acting to reduce the industry’s emissions under a global common framework has allowed the saving of billions of dollars to the group [54].

Analogously, the Environmental Costa Rican policies, as its Payment for Ecosystem Services program, which has been facing financial shortcomings [55], could have their financial sustainability enhanced with the consideration of their positive global externalities. The efficacy of the policy would rely pretty much on an adequate setting of compensation between Costa Rica and other countries. This is not as simple as put here; on the contrary, it is complex and time demanding. Indeed, many discussions of the last COP^7^ revolved around Paris Agreement’s Article 6, which in general stands for voluntary cooperation between international actions. Transparency, quality of GHG reductions and timeframes were stated as some of the most daring challenges looming ahead.

## Conclusion

Climate crises should be addressed from a global perspective, following the standing feature of climate change. Current policies are still unable to attend such claims, and economic analyses usually consider only local impacts from climate interventions. Nonetheless, it is urgent to acknowledge that the atmosphere is a global public good and that GHG effects respect no boundaries. Thus, avoided emissions generate economic benefits that are global and should not be ignored from climate planning.

Furthermore, financing approaches such as the NAMA could further leverage the transition to a green economy within the agricultural and livestock sector, as it seems to have the potential to concomitantly generate a negative carbon-balance in addition to positive socio-economic outcomes—profits, investments, jobs.

The CBA in this paper included the so-called global positive externalities of sectorial climate policies, where avoided emissions from a climate policy in the Costa Rican cattle sector were accounted for their potential for generating global benefits. Such an approach showed that a national sectorial climate policy, which had already presented positive private perspectives, may become more attractive when positive externalities are added, particularly within long-term scenarios.

The core question is that, even under efficient global economic perspectives, climate policies are likely to be dismissed due to costs remaining confined to a singular (or a group of) agent(s). Although far from being a silver bullet, adding global positive externalities to the expected public and private benefits of a sectorial policy can be an effective way to enhance the rationality backing climate investments, particularly in developing regions.

## Data Availability

Further data and information can be found at Dall’Orsoletta [46] (in Portuguese), which served as a primary base for the present paper. The original work and database that gave base to Dall’Orsoletta [46], and the datasets generated and/or analysed during the current study, are available from the corresponding author on reasonable request.

## Appendices

### Appendix 1

Further detailed information on NAMA program.

**Table:**

Investments, costs and number of farms applying the measures						
Farm		Initial investment (USD/ha)	Saving (USD/ha)	Operational costs (USD/ha/year)	Covered area by technology (ha/farm)^a^	Farms implementing the measures
*Rational grazing* + *hedges*						
Meat		1,027	n/a	27	13.58	1,004
Milk		1,027	n/a	27	2.7	318
Mixed		1,027	n/a	27	15.09	478
*Enhanced selection of pastures*						
Meat		495	n/a	74	5.43	1,004
Milk		495	n/a	74	1.35	318
Mixed		495	n/a	74	9.43	478
*Fertilization improvements*						
Meat		(not considered for meat farms)				
Milk		340	449	681	13.52	318
Mixed		340	0	0	18.86	478

^a^Pasture area's fraction in which each technology will be applied. Data from Dall’Orsoletta [46]

**Table:**

Average productive gains considered				
Measures	Meat farms	Milk farms	Mixed farms	
	(ton alive animal/year)	(ton milk/year)	Meat (ton alive animal/year)	Milk (ton milk/year)
Rational grazing + hedges	150	5,104	151	979
Enhanced selection of pasture	150	475	154	428
Fertilization improvements	n/a	1,415	n/a	447

**Table:**

Expected financial gains according to farms' purpose and measures (USD/ha)									
Year	No. farms	Meat		Milk			Mixed		
		RG + H	EP	RG + H	EP	FI	RG + H	EP	FI
1	91	255	255	1,894	176	525	619	419	166
2	253	264	264	1,959	182	543	640	434	172
3	577	273	273	2,031	189	563	663	450	178
4	1,014	278	278	2,068	192	573	676	458	181
5	1,800	280	280	2,08	193	577	679	461	182
6	1,800	283	283	2,101	195	583	686	465	184
7	1,800	287	287	2,13	198	591	696	472	187
8	1,800	291	291	2,164	201	600	707	479	190
9	1,800	295	295	2,192	204	608	716	485	192
10	1,800	298	298	2,213	206	614	723	490	194
11	1,800	300	300	2,23	207	618	729	494	195
12	1,800	302	302	2,247	209	623	734	498	197
13	1,800	305	305	2,265	211	628	740	501	199
14	1,800	307	307	2,282	212	633	745	505	200
15	1,800	309	309	2,296	213	637	750	508	201

RG: Rational grazing + hedges, EP: Enhanced selection of pasture, FI: Fertilization improvements

### Appendix 2

Details of opportunity cost harmonization and aggregation.

**Table:**

Opportunity costs details			
Input		Biogas generation	Concentrate feedings^a^
Mitigation potential	kg CO_2_/animal/year	500.23	128.22
Total costs	USD/animal	41.55	81.05
Net Costs	USD/animal	− 24.58	− 15.52
Associated economic benefits	USD/animal	66.13	96.57

^a^Mean values. From Sapkora et al. [49]

Equalization of the OC into NAMA features through a linear function^a^.

**Table:**

Sapkota et al. (2019)		NAMA		Harmonization index equation (x)
(a) Benefits (AE + OG)	93,061,040	(c) Benefits (AE + OG)	247,364,193	[(d*a)/c]/d = x
(b) Costs (100%)	52,273,494	(d) Costs (100%)	42,167,568	x = 0.303

^a^As an example, the equalization for the Nordhaus 15-year Social (SN 15) scenario is shown. AE: avoided emissions, OG: operational gains.

Harmonization of the OC for Nordhaus 15-year Social analyses, with the harmonization index.

**Table:**

Inputs	OC (USD)	Equation
(a) Sapkota et al. (2019)	40,787,546	(AE + OG)-NC
(b) Harmonized	12,236,264	b = a*x

E: avoided emissions, OG: operational gains, NC: net costs. Data from Dall’Orsoletta [46] and Sapkota et al. [49]

### Appendix 3

Detailed table with calculations for scenario SS 15C.

## Footnotes

1 Hereafter, climate will always be used within the climate change context, not the climate itself.

2 Guterres, A., 2020. A Time to Save the Sick and Rescue the Planet (adapted). The New York Times, 28/Apr/2020: https://www.nytimes.com/2020/04/28/opinion/coronavirus-climate-antonio-guterres.html?smid=nytcore-ios-share.

3 The Costa Rican Nationally Determined Contribution (NDC) under the Paris Agreement: https://www4.unfccc.int/sites/ndcstaging/PublishedDocuments/Costa%20Rica%20First/INDC%20Costa%20Rica%20Version%202%200%20final%20ENG.pdf.

4 A clear and exact definition for the term “avoided emissions” is still open to debate [41]. This paper uses the term to refer to the amount of GHG that would not be released into the atmosphere as a result of operational improvements within the referred cattle’s value chain.

5 Dall’Orsoletta [46] had access to this database (from personal communication) and made it available.

6 Considering the large diversity in cattle management practices throughout the world, and the difficulty in finding cases in Costa Rica that could serve as a basis for the OC, the authors found that Sapkota et al. [49] was the best feasible basis for establishing the OC. The Indian study approaches the same mitigation measures as the ones in this paper, and from a climate-related perspective. Moreover, it was equally modelled for the cattle sector of a developing tropical country.

7 The 25^th^ Conference of the Parties of the United Nations Framework Convention on Climate Change.
